# Gastrointestinal Dysfunction in Chinese Patients with Parkinson's Disease

**DOI:** 10.1155/2019/3897315

**Published:** 2019-08-20

**Authors:** Xiaoling Qin, Xue Li, Zai'e Xin, Zaili Li

**Affiliations:** ^1^Department of Neurology, Xuzhou Central Hospital, Xuzhou Clinical School of Xuzhou Medical University, 199 Jiefang Road, Xuzhou, Jiangsu Province 221009, China; ^2^Xuzhou Medical University, 209 Tongshan Road, Xuzhou, Jiangsu Province 221002, China

## Abstract

**Purpose:**

To observe the occurrence and influencing factors of the symptoms related to the digestive system of people at the early and middle stages of PD and of healthy controls (HCs) using a questionnaire.

**Method:**

The questionnaire was given to 108 PD patients at early and middle stages. Twelve symptoms related to the digestive system, of which seven were listed on the Parkinson's Disease Non-Motor Symptom Scale (PD-NMSS) and the Scale for Outcomes in Parkinson's disease-Autonomic (SCOPA-AUT) (dysgeusia, dysphagia/choking, salivation, early satiety, constipation, loose stools, and fecal incontinence) and five symptoms used in the diagnosis and treatment of PD (loss of appetite, dry mouth, mouth pain, nausea and vomiting), were used. The questionnaire was also given to HCs.

**Results:**

There was no significant difference in age, sex, height, weight, or body mass index (BMI) between the PD group and HCs. Of the 108 people at the early and middle stages of PD, the most common symptoms related to the digestive system were 64 cases of dry mouth (59.26%), 53 cases of constipation (49.07%), and 40 cases of dysgeusia (37.04%). Multivariate binary logistics regression revealed that dysgeusia (*P* < 0.001), dysphagia (*P* = 0.004), early satiety (*P* = 0.001), and constipation (*P* = 0.007) were more likely to occur in males. BMI, disease duration, and motor symptoms had no significant correlation with the symptoms related to the digestive system (*P* > 0.05 for all).

**Conclusions:**

Dry mouth, constipation, dysgeusia, loss of appetite, early satiety, and dysphagia are the most common (and possibly characteristic symptoms) related to the digestive system in people at the early and middle stages of PD. Being male is a risk factor for dysgeusia, dysphagia, early satiety, and constipation.

## 1. Introduction

Parkinson's disease (PD) is the second most prevalent neurodegenerative disease that impairs the health of middle-aged and elderly people, and PD prevalence increases with age [1]. The nonmotor symptoms of PD include neuropsychological symptoms, autonomic dysfunction, sleep disturbance, and sensation disorders. However, gastrointestinal dysfunction is the most common symptom manifested in all stages of PD [2].

The cause of PD is not known. However, over the past decade, studies have suggested bidirectional signaling between the brain and gastrointestinal tract (GIT) (i.e., brain-gut axis) in PD and the occurrence and development of PD have garnered wide attention [3].

We observed gastrointestinal dysfunction and symptoms in the digestive system of PD patients via questionnaires to explore the influencing factors.

## 2. Patients and Methods

### 2.1. Ethical Approval of the Study Protocol

The study protocol was approved by the Biomedical Research Ethics Committee of Xuzhou Central Hospital (XCH; Xuzhou, China).

### 2.2. Inclusion Criteria

The inclusion criteria were as follows: patients who met the latest diagnostic criteria for PD developed by the Movement Disorder Society [4] at Hoehn–Yahr (H–Y) stage I–III and who had not undergone surgery or deep-brain stimulation.

### 2.3. Exclusion Criteria

The exclusion criteria were as follows: patients with Parkinsonism-plus syndrome, secondary PD, cardiovascular diseases, psychological diseases, diseases of the digestive system, and active infection (e.g., tuberculosis) or who were taking drugs in the past 3 months that may affect gastrointestinal function.

### 2.4. Study Cohort

Data for 108 PD patients examined at XCH from May 2016 to May 2018 were collected from their electronic medical records within the XCH database.

The control group comprised 76 healthy controls (HCs) examined in the Physical Examination Center of XCH during the same period and matched with the PD group with respect to age, sex, level of education, and height.

### 2.5. Data Collection

General data (age, sex, level of education, height, and weight) were collected first, followed by the age at disease onset, disease duration, and classification of motor symptoms (which were assessed using the H–Y scale). Then, questions regarding symptoms in the digestive system were focused upon. Questionnaires were administered for 12 symptoms. Seven of these symptoms were listed on Parkinson's Disease Non-Motor Symptom Scale (PD-NMSS) [5] and the Scale for Outcomes in Parkinson's disease-Autonomic (SCOPA-AUT) [6]: dysgeusia, dysphagia/choking, salivation, early satiety, constipation (stool frequency <2 times/week), loose stools, and fecal incontinence. The other five symptoms were related to the digestive system used commonly for the diagnosis and treatment of PD: loss of appetite, dry mouth, mouth pain, nausea, and vomiting. The data collection and symptom enquiries mentioned above were also carried out for HCs.

### 2.6. Statistical Analyses

SPSS 22.0 (IBM, Armonk, NY, USA) was used for data analyses. Measurement data are the mean ± standard deviation. Count data are given as percentages. The independent *t*-test was employed for conducting intergroup comparison of measurement data. The *χ*^2^ test was used for count data. A multivariate binary logistic regression model was used to analyze the risk factors of symptoms related to the digestive system of PD patients. *P* < 0.05 was considered significant.

## 3. Results

### 3.1. General Data of the Study Cohort and HCs

Of the 108 PD patients, 59 (53.91%) were male and 49 (46.09%) were female. The mean age was 67.97 ± 8.58 years (Table 1). The mean duration of PD was 5.03 ± 4.87 years. 65 (60.19%), 26 (24.07%), and 17 (15.74%) cases received education of primary school and lower, middle school, and college, respectively. In addition, 38 cases (35.19%) had slow movements (bradykinesia) and rigidity or instability in posture. Also, 70 (64.81%) patients had tremors and slow movements with/without rigidity or instability in posture. In addition, 20 (18.52%) patients were H–Y stage I, 55 (50.93%) stage II, and 33 (30.56%) stage III.

### 3.2. Symptoms Related to the Digestive System of the PD Group and HCs

In the PD group, the common symptoms related to the digestive system were 64 (59.26%) cases of dry mouth, 53 cases (49.07%) of constipation, and 40 cases (37.04%) of dysgeusia (Table 2). Less common symptoms were 35 (32.41%) cases of loss of appetite and 26 (24.07%) cases of early satiety. The least common symptoms were 20 (18.52%) cases of dysphagia, 11 (10.19%) cases of nausea, 10 (9.26%) cases of salivation, 7 (6.48%) cases of mouth pain, 4 (3.70%) cases of vomiting, and 1 case (0.87%) of loose stools.

In HCs, there were 13 (17.11%) cases of dysgeusia, 6 (7.89%) cases of early satiety, 6 (7.89%) cases of loss of appetite, 5 (6.58%) cases of nausea, 3 (3.95%) cases of salivation, and 1 (1.32%) case of mouth pain.

Compared with HCs, patients in the PD group were more likely to have symptoms related to the digestive system, including dry mouth (*P* < 0.001), dysgeusia (*P* < 0.003), dysphagia (*P* < 0.001), loss of appetite (*P* < 0.001), early satiety (*P*=0.004), and constipation (*P* < 0.001). The significant difference between the groups for these symptoms suggested that these symptoms were more pronounced at the early and middle stages of PD. No significant difference was observed between the two groups for the prevalence of nausea (*P*=0.372), salivation (*P*=0.166), mouth pain (*P*=0.091), vomiting (*P*=0.09), or loose stools (*P*=0.4).

### 3.3. Factors Influencing the Symptoms Related to the Digestive System in the PD Group

Multivariate binary logistics regression was carried out to analyze the risk factors for the six symptoms related to the digestive system with a significant difference in Table 2 (dry mouth, dysgeusia, dysphagia, loss of appetite, early satiety, and constipation) (Table 3). Dysgeusia (*P* < 0.001), dysphagia (*P*=0.004), early satiety (*P*=0.001), and constipation (*P*=0.007) were more likely to occur in males. Body mass index (BMI), disease duration, and motor symptoms had no significant correlation with the symptoms related to the digestive system (*P* > 0.05 for all).

## 4. Discussion

The nonmotor symptoms of PD are seen commonly in the clinic, but their classification is not straightforward. A common practice is to divide them into five categories: cognitive dysfunction, neuropsychiatric disorder, autonomic dysfunction, sleep disturbance, and other nonmotor symptoms. Assessment of the nonmotor symptoms of PD is done primarily using PD-NMSS [5], SCOPA-AUT [6], and the revised Unified Parkinson's Disease Rating Scale-I [7]. Impairment of the autonomic nervous system is manifested primarily by GIT dysfunction: salivation, constipation, dysphagia, and nausea [8]. Some recent studies have paid attention to oral health and dysgeusia in PD patients [9, 10].

The human digestive system consists of the GIT plus the accessory organs of digestion (tongue, salivary glands, pancreas, liver, and gallbladder) [11]. By focusing on the symptoms related to the digestive system, one might be able to describe the symptoms that aid description of the nonmotor symptoms of PD.

We administered a questionnaire and undertook statistical analyses for people at the early and middle stages of PD. We concentrated on 12 symptoms related to the digestive system (seven in PD-NMSS and SCOPA-AUT, and five other commonly observed symptoms used for the diagnosis and treatment of PD).

The most common symptoms related to the digestive system of PD patients were dry mouth, constipation, and dysgeusia. Dry mouth had the highest prevalence (59.26%), significantly higher than that in HCs (*P* < 0.001). Barbe and colleagues showed a prevalence of 50% for dry mouth and correlation with the classification of PD symptoms, age, drug administration, food intake, dysphagia, and hyposalivation [12]. The prevalence of dry mouth in our study was slightly higher than that observed in other studies. This difference might have been due to the definition of dry mouth and medications administered (especially anticholinergic agents). Constipation is one of the most common nonmotor symptoms of PD patients. Constipation can occur 20 years before the motor symptoms of PD become apparent, with a prevalence as high as 24–70% [13, 14]. In the present study, the prevalence of constipation was 55.65%, significantly higher than that in HCs (*P* < 0.001), and similar results have been noted by other scholars. Constipation is related to the reduced water intake, lack of movement, and ageing of PD patients and, more importantly, to a decrease in colonic transit capability [15]. Pfeiffer and colleagues noted that the colonic transit time of PD patients was twofold less than that of persons in a control group [16]. Studies have shown accumulation of Lewy bodies in the vasoactive intestinal peptide-ergic neurons of the intrinsic nervous system, which suggests that loss of inhibitory motor neurons will cause impaired reflex relaxation of distal smooth muscles and, consequently, leads to slow colonic transit [15]. Intestinal tissue is more readily available than brain tissue, so constipation is expected to be a biomarker at the prodromal stage of PD and to play a part in the early diagnosis of PD [13].

Few studies have focused on dysgeusia in PD patients. In the present study, the prevalence of dysgeusia in the PD group was 37.39%, significantly higher than that in HCs (*P*=0.003). Cecchin and colleagues found that PD patients had significantly diminished taste [10]. Though the relationship between dysgeusia and PD is not clear, olfactory dysfunction is a prodromal symptom of PD and could become an early biomarker of PD [17]. Using 15-year follow-up, a cohort study of 474 patients with idiopathic smell/taste loss revealed that 28.6% of patients who had both olfactory dysfunction and dysgeusia eventually developed PD, whereas only 9.9% of patients with olfactory dysfunction only and 3.8% of patients with dysgeusia only eventually developed PD, suggesting that taste assessment and olfactory assessment are equally important [18]. In the present study, dysgeusia was a subjective complaint of patients and was not measured objectively or quantitatively. In future studies, it will be necessary to confirm our results via quantitative methods.

Gastroparesis (i.e., delayed gastric emptying) is associated with a decrease in gastric mobility that eventually affects intestinal transport [19]. Gastroparesis may be associated with degeneration of neurons in the muscular plexus and brainstem, with the clinical manifestations of early satiety, satiety, nausea, vomiting, loss of weight, abdominal pain, and abdominal distension [19]. In the present study, the prevalence of early satiety, nausea, and vomiting in PD patients was 24.07%, 10.19%, and 3.70%, respectively. There was a significant difference (*P*=0.004) in early satiety between the PD group and HCs. Therefore, early satiety might be a characteristic symptom of the early and middle stages of PD, whereas nausea and vomiting might be associated only with anti-PD drugs (mostly dopamine receptor agonists) [20]. Intestinal absorption of L-DOPA may be slowed down due to a prolonged gastric retention time, thereby reducing its therapeutic effect and hindering improvement of motor symptoms [21]. Therefore, paying attention to gastroparesis-associated PD is useful.

Dysphagia is another pathophysiological feature of PD. Often, dysphagia is caused by slow movements and weakened control of tongue movements and may also be caused (at least in part) by direct injury to the intrinsic nervous system [22]. Varanese and colleagues showed that up to 50% of PD patients may have swallowing problems, which occur mainly in the late stage of PD [23]. The prevalence of dysphagia in early-stage and middle-stage PD in our study was 21.74%, much lower than that in late-stage PD.

In recent years, although researchers have studied the conditions of oral health or dental health of PD patients, studies have been sparse and the results controversial [9, 24]. Tooth loss, dental caries, gingival atrophy, and increased tooth movement occur in PD patients [25]. “Burning mouth syndrome” (BMS) is a group of symptoms manifested mainly by the sensation of burning pain in the oral mucosa. Studies have shown that BMS is very common in patients with PD. This might be related to a decrease in the dopamine level and disorder of DOPA regulation [26]. Different from the results of other studies, the prevalence of oral pain in PD patients in our study was 8.70% and our study did not show significance in comparison with that in HCs. More studies are needed to explore the relationship between oral health and the occurrence and development of PD.

Risk analysis undertaken by introducing related factors such as age, sex, BMI, disease duration, and motor symptoms into logistic regression showed that being male was a risk factor for the occurrence of dysgeusia, dysphagia, early satiety, and constipation in early- and middle-stage PD. Other factors had no significant influence on the symptoms related to the digestive system. Epidemiological data have shown that being male has a higher risk of PD than being female [1, 27]. There was also a sex-based difference in motor and nonmotor symptoms in PD patients. Compared with male patients, female patients are more likely to have cardiovascular events, fatigue, and weakness [28] and more prone to emotional, sleep, and pain problems [29]. Male PD patients are more likely to have sexual dysfunction, depression, [28] and urinary symptoms [29]. Few studies have been conducted on sex-based differences in PD, and the conclusions have been inconsistent. Therefore, high-quality, prospective, and longitudinal studies are required to investigate sex-based differences in PD.

## 5. Conclusions

Dry mouth, constipation, dysgeusia, loss of appetite, early satiety, and dysphagia were the most common symptoms related to the digestive system in PD patients. Being male was a risk factor for constipation, dysgeusia, dysphagia, and early satiety. Symptoms related to the digestive system may affect the digestion as well as the absorption of food and drugs and, thus, may further affect the occurrence and development of PD. Therefore, attention should be paid to active treatment of these symptoms and exploration of their mechanism of action.

## Figures and Tables

**Table 1:** Comparison of general data between the PD group and HCs.

	PD group (*n* = 108)	HCs (*n* = 76)	*χ* ^2^	*P*
Age (years)	67.97 ± 8.58	66.24 ± 7.92	1.403	0.161
Male/female (cases)	59/49	41/35	0.008	0.927
Height (cm)	162.96 ± 7.14	163.56 ± 6.74	−0.581	0.562
Weight (kg)	64.84 ± 10.47	66.91 ± 11.25	−1.264	0.208
BMI (kg/m^2^)	24.35 ± 3.14	25.11 ± 3.63	−1.477	0.142
PD duration (years)	5.03 ± 4.87	—		
Education				
Primary school and lower	65 (60.19%)	43 (56.57%)	0.239	0.887
Middle school	26 (24.07%)	20 (26.31%)		
College	17 (15.74%)	13 (17.11%)		
Motor symptoms				
Bradykinesia with/without rigidity or instability in posture	38 (35.19%)	—		
Tremors + bradykinesia with/without rigidity or instability in posture	70 (64.81%)	—		
H-Y stage				
I	20 (18.52%)	—		
II	55 (50.93%)	—		
III	33 (30.56%)	—		

**Table 2:** Occurrence of symptoms related to the digestive system of the PD group and HCs (%).

Symptoms related to the digestive system	PD group (*n* = 108)	HCs (*n* = 76)	*χ* ^2^	*P*
Mouth pain	7 (6.48%)	1 (1.32%)	2.862	0.091
Dry mouth	64 (59.26%)	0 (0)	69.05	<0.001
Dysgeusia	40 (37.04%)	13 (17.11%)	8.642	0.003
Dysphagia	20 (18.52%)	0 (0)	15.79	<0.001
Early satiety	26 (24.07%)	6 (7.89%)	8.128	0.004
Loss of appetite	35 (32.41%)	6 (7.89%)	16.01	<0.001
Nausea	11 (10.19%)	5 (6.58%)	0.796	0.372
Salivation	10 (9.26%)	3 (3.95%)	1.917	0.166
Vomiting	4 (3.70%)	0 (0)	2.977	0.09
Constipation	53 (49.07%)	0 (0)	52.39	<0.001
Loose stool	1 (0.87%)	0 (0)	0.708	0.4
Fecal incontinence	0 (0)	0 (0)	—	—

**Table 3:** Factors influencing some of the symptoms related to the digestive system.

	Beta	Wald	*P*	OR	95%CI
Dry mouth					
Sex	−0.142	0.111	0.74	0.868	0.377–2.000
Age	−0.002	0.005	0.942	0.998	0.948–1.051
BMI	−0.032	2.187	0.139	0.969	0.929–1.010
Disease duration	0.04	0.778	0.378	1.041	0.952–1.139
Motor symptoms	−0.657	2.309	0.129	0.518	0.222–1.201
Dysgeusia					
Sex	−3.189	23.155	<0.001	0.041	0.011–0.151
Age	0.02	0.384	0.535	1.02	0.958–1.086
BMI	0.027	0.897	0.344	1.027	0.972–1.087
Disease duration	0.024	0.146	0.702	1.024	0.908–1.155
Motor symptoms	−0.322	0.396	0.529	0.725	0.266–1.973
Dysphagia					
Sex	−3.131	8.481	0.004	0.044	0.005–0.359
Age	−0.002	0.002	0.967	0.998	0.929–1.073
BMI	−0.021	0.365	0.546	0.979	0.914–1.049
Disease duration	0.038	0.261	0.609	1.039	0.898–1.202
Motor symptoms	0.249	0.182	0.669	1.282	0.410–4.014
Early satiety					
Sex	−3.543	10.918	0.001	0.029	0.004–0.237
Age	0.009	0.063	0.802	1.009	0.941–1.082
BMI	0.016	0.242	0.623	1.016	0.954–1.083
Disease duration	−0.029	0.137	0.711	0.972	0.835–1.131
Motor symptoms	0.172	0.099	0.753	1.188	0.406–3.479
Loss of appetite					
Sex	0.313	0.479	0.489	1.368	0.563–3.320
Age	−0.012	0.18	0.672	0.988	0.935–1.044
BMI	0.041	3.653	0.058	1.042	1.010–1.089
Disease duration	−0.024	0.26	0.61	0.977	0.892–1.070
Motor symptoms	0.749	2.498	0.114	2.114	0.835–5.351
Constipation					
Sex	−1.165	7.224	0.007	0.312	0.133–0.729
Age	0.016	0.356	0.551	1.016	0.965–1.070
BMI	0.001	0.002	0.965	1.001	0.960–1.044
Disease duration	−0.006	0.046	0.9	0.994	0.908–1.089
Motor symptoms	−0.531	1.546	0.214	0.588	0.255–1.358

## Data Availability

The data used to support the findings of this study are included within the article. Requests for data, 12 months after publication of this article, will be considered by the corresponding author.
